# Extrachromosomal circular DNAs in prostate adenocarcinoma: global characterizations and a novel prediction model

**DOI:** 10.3389/fphar.2024.1464145

**Published:** 2024-09-17

**Authors:** Qingliu He, Qingfu Su, Chengcheng Wei, Pu Zhang, Weihui Liu, Junyi Chen, Xiaoping Su, Wei Zhuang

**Affiliations:** ^1^ Department of Urology, The Second Affiliated Hospital of Fujian Medical University, Quanzhou, China; ^2^ Department of Urology, The First Affiliated Hospital of Chongqing Medical University, Chongqing, China; ^3^ Department of Urology, Sichuan Provincial People’s Hospital, School of Medicine, University of Electronic Science and Technology of China, Chengdu, China; ^4^ Department of Nursing, The Second Affiliated Hospital of Fujian Medical University, Quanzhou, China

**Keywords:** prostate adenocarcinoma, extrachromosomal circular DNA, risk model, immune infiltration, immunotherapy

## Abstract

**Background:**

The role of focal amplifications and extrachromosomal circular DNA (eccDNA) is still uncertain in prostate adenocarcinoma (PRAD). Here, we first mapped the global characterizations of eccDNA and then investigate the characterization of eccDNA-amplified key differentially expressed encoded genes (eKDEGs) in the progression, immune response and immunotherapy of PRAD.

**Methods:**

Circular_seq was used in conjunction with the TCGA-PRAD transcriptome dataset to sequence, annotate, and filter for eccDNA-amplified differentially expressed coding genes (eDEGs) in PRAD and para-cancerous normal prostate tissues. Afterwards, risk models were created and eKDEGs linked to the PRAD prognosis were identified using Cox and Lasso regression analysis. The immune microenvironment of the risk model was quantified using a variety of immunological algorithms, which also identified its characteristics with regard to immunotherapy, immune response, and immune infiltration.

**Results:**

In this research, there was no significant difference in the size, type, and chromosomal distribution of eccDNA in PRAD and para-cancerous normal prostate tissues. However, 4,290 differentially expressed eccDNAs were identified and 1,981 coding genes were amplified. Following that, 499 eDEGs were tested in conjunction with the transcriptome dataset from TCGA-PRAD. By using Cox and Lasso regression techniques, ZNF330 and PITPNM3 were identified as eKDEGs of PRAD, and a new PRAD risk model was conducted based on this. Survival analysis showed that the high-risk group of this model was associated with poor prognosis and validated in external data. Immune infiltration analysis showed that the model risks affected immune cell infiltration in PRAD, not only mediating changes in immune cell function, but also correlating with immunophenotyping. Furthermore, the high-risk group was negatively associated with anti-*CTLA-4*/anti-*PD-1* response and mutational burden. In addition, Tumor Immune Dysfunction and Exclusion analyses showed that high-risk group was more prone to immune escape. Drug sensitivity analyses identified 10 drugs, which were instructive for PRAD treatment.

**Conclusion:**

*ZNF330* and *PITPNM* are the eKDEGs for PRAD, which can be used as potential new prognostic markers. The two-factor combined risk model can effectively assess the survival and prognosis of PRAD patients, but also can predict the different responses of immunotherapy to PRAD patients, which may provide new ideas for PRAD immunotherapy.

## 1 Introduction

Prostate adenocarcinoma (PRAD) is the second most common cancer and the fifth cause of cancer deaths in men throughout the world (Sung et al., 2021), with its incidence increasing by 3% per year since 2014 (Siegel et al., 2024). According to statistics, in the United States, PRAD cases will be responsible for 11% of all fatalities and 29% of all male cancer cases by 2024 (Siegel et al., 2024). Despite the availability of several treatment options, including androgen restriction, surgery, radiation, chemotherapy, and endocrine therapy, 20%–30% of cases of PRAD advance to metastatic prostate cancer (mPCa) (Sandhu et al., 2021) which ultimately develops into metastatic prostate cancer that is resistant to denudation (mCRPC) until death. The emerging research in immunotherapy holds great promise for improving the lives of PRAD patients (Rebello et al., 2021). Recent data indicates that the tumour microenvironment (TME) has a major role in determining the prognosis of PRAD (Xu et al., 2022). A better prognosis for patients and enhanced immunological control of PRAD are linked to immune infiltration in the TME (Fridman et al., 2012). Immunotherapies, including immune checkpoint inhibitors (ICIs) and chimeric antigen receptor T-cell therapies, can improve anti-tumour outcomes and overall survival (OS) in patients with advanced PRAD (Abida et al., 2019). Immunotherapy for PRAD has made great progress in recent years. A mendelian randomisation study has provided evidence for a causal relationship between immune cells and PRAD, with important implications for clinical diagnosis and treatment (Ye et al., 2024). In addition, Recent studies have highlighted the potential of PD-1 and PD-L1 inhibitors in treating metastatic castration-resistant prostate cancer (Antonarakis et al., 2020; Philippou et al., 2020). New vaccine strategies have emerged, building on the success of sipuleucel-T (Sutherland et al., 2021). Recent trials have explored vaccines targeting prostate-specific antigens (PSA) (Lopez-Bujanda et al., 2021). Furthermore, advances in cell-based therapies, such as chimeric antigen receptor (CAR) T-cell therapy (Narayan et al., 2022) and tumor-infiltrating lymphocytes (TILs) (Kaur et al., 2022), have been reported. But only a small percentage of mPCa patients respond to immunotherapies (Abida et al., 2019) for the main possible reason that PRAD is an immunocold tumour with defective tumour suppression and poor immune infiltration (Melo et al., 2021). Consequently, it is critical to look for novel biomarkers, targets, and characteristics in order to develop fresh treatment approaches for breaking through the immunotherapeutic obstacles associated with PRAD.

The unique topology and genetic characteristics of extrachromosomal circular DNA (eccDNA), a circular DNA derived from chromosomes that may be chromosome-independent (Hotta and Bassel, 1965), have led to new understandings of cancer surveillance, diagnosis, treatment, and prediction. EccDNA has been implicated in the development and progression of cancer (Ling et al., 2021). For example, Turner KM et al. (Turner et al., 2017) demonstrated that eccDNAs could act as enhancer elements to mediate overexpression of oncogenes and amplify more copies of oncogenes. Andrisani O et al. (Andrisani, 2024; Zou et al., 2024) found that eccDNAs acted as miR-17–92 amplicons in hepatocellular carcinomas (HCCs), which is a risk factor for poor prognosis of patients. In addition, eccDNA is frequently found in a variety of cancers (Chen et al., 2024a), including PRAD.

Increasing evidence has revealed the immunostimulatory activity of eccDNA in tumours, as well as its route and possible therapeutic implications in the immune response (Wang et al., 2021). For example, Ying Zhang et al. (Zhang et al., 2023) found that risk models generated by eccDNA-amplified encoded genes (eGenes) may affect the prognosis of ovarian cancer patients by modulating some immune cells or immune checkpoints, suggesting that eGenes are important factors in the immune infiltration and immune response of tumour cells.

However, the expression profile of eccDNA in PRAD has received little attention. Although Chen JP et al. (Chen JP et al., 2024) and Luo X et al. (Luo et al., 2023) have identified the potential of eccDNA in the diagnosis of PRAD, it is not clear whether there are specific eccDNAs that are exclusively involved in the immune response to PRAD. Therefore, in this study, in this investigation, we developed a novel risk model based on eKDEGs in PRAD, which was tested by sequencing eccDNA from PRAD and paracancerous normal prostate tissues with the TCGA-PRAD transcriptome dataset. We investigate the predictive features and their involvement in immune infiltration and immune response, with the goal of discovering new biomarkers and therapeutic targets for PRAD immunotherapy.

## 2 Methods

### 2.1 Tissue specimen collection

This experimental study was approved by the Ethics Committee of our hospital, and three cases of patients with limited prostate adenocarcinoma were collected from the Department of Urology of our hospital in the year of 2022, under the guidance of the physicians of the Department of Pathology. The PRAD tumour tissue specimens were used as the tumour group, and the paracancerous normal prostate tissue specimens were used as the normal group. Tissue specimens were collected and stored in liquid nitrogen to send for eccDNA sequencing.

### 2.2 eccDNA sequencing

PRAD and paracancerous normal prostate tissue specimens were subjected to eccDNA sequencing assisted by CloudSeq Biotech Inc. (Shanghai, China) using the circle-seq (Møller et al., 2018) method. Briefly, cell deposits were resuspended in L1 buffer (Plasmid Mini AX; A&A Biotechnology) supplemented with protease K (ThermoFisher) prior to digestion at 50°C overnight. Digested samples were alkali-treated and column-purified by following the instructions of the Plasmid Mini AX kit. Column-purified DNA samples were digested by FastDigest MssI (ThermoFisher) at 37°C for 16 h to remove mitochondrial circular DNA. Then, the samples were incubated with Plasmid-Safe ATP-dependent DNase (Epicentre) at 37°Cfor 1 week to remove the remaining linear DNA. The samples were then supplemented with 30U of DNase and a proportional amount of ATP every 24 h. The treated samples were used as templates for eccDNA amplification by using the RCA DNA Amplification Kit (GenSeq Inc.), followed by purification with the MinElute Reaction Cleanup Kit (Qiagen). Library preparation of purified DNA was performed with the GenSeq^®^ Rapid DNA Lib Prep Kit (GenSeq Inc.). High-throughput sequencing was performed on an Illumina NovaSeq 6000 sequencer in 150 bp double-ended mode to obtain the raw data. Quality control was performed with Q30 as following sequence, low-quality reads were removed firstly by using cutadapt software (v1.9.1), and high-quality clean reads were aligned to the reference genome by using bwa software (v0.7.12). Next, all eccDNAs were identified with circle-map software (v1.1.4) and then raw soft-clipped read counts of the break point were obtained by using SAMtools (v1.9) software. Normalisation and differential analysis were performed by using DESeq2 [7] (v1.38.3) software. Annotation of eccDNA was performed by using bedtools software (v2.27.1) and enrichment analyses were performed by using the eDEGs. eccDNA visualisation was performed by using IGV (v2.4.10) software.

### 2.3 Analysis of TCGA-PRAD dataset

Transcriptional profiles, clinical features, tumour mutation burden (TMB) and microsatellite instability (MSI) scores of PRAD were downloaded from The Cancer Genome Atlas Program (TCGA, https://portal.gdc.cancer.gov/) database. Validation set data were obtained from cBioPortal-SU2C/PCF (https://www.cbioportal.org/) and GEO70770 (https://www.ncbi.nlm.nih.gov/geo/). Data preprocessing and DEGs analysis were perfoemed by the “limma” and “affay” packages in the R environment. The coding genes amplified by differential eccDNA were taken to intersect with the DEGs of TCGA-PRADt to obtain eccDNA-amplified differentially expressed coding genes (eDEGs). Gene Ontology (GO), Kyoto Encyclopedia of Genes and Genomes (KEGG) functional enrichment analyses were performed using the “clusterProfiler” package. GO enrichment analysis described the potential functions of genes in terms of Molecular Function (MF), Cellular Component (CC) and Biological Process (BP). KEGG analysed the major metabolic and signal transduction pathways in which the genes were involved through pathway annotation. Cox regression analysis and Lasso analysis were used to further identify prognostic genes and construct risk models. Cox regression analysis was performed using the “survival” package and Lasso analysis was performed using the “glmnet” package. Kaplan-Meier (KM) survival analysis plots and risk factor association plots based on the risk models were constructed using the “survival” and “ggplot2” packages. Nomograms were used to visualise the 1-year, 3-year, and 5-year survival predictions of the risk model. Time-dependent ROC curves were used to verify the model accuracy. Gene Set Enrichment Analysis (GSEA) was performed using the “Cluster Profiler,” “org. Hs.eg.db”, and “enrichplot” package to identify biological processes and enrichment pathways of the key gene. Quantification of the immune microenvironment was performed using XCELL, MCPCOUNTER, CIBERSORT, TIMER, EPIC, and QUANTISEQ algorithms. Identify the characteristics and differences in immune infiltration (performed with the “CIBERSORT” and “reshape2” packages), immune function (performed with the “RColorBrewer” package), and immune subtypes (performed with the “RColorBrewer” package) of risk models based on quantitative immune microenvironment results. ESTIMATE analysis identified specific signals associated with stromal and immune cell infiltration in tumour tissue and predicted the level of infiltrating stromal and immune cells by calculating stromal and immune scores, which were performed with the “utils” package. Gene mutation frequency and mutation burden of the risk models were analyzed with the“maftools” package. Immunophenotype score (IPS) was obtained from The Cancer Immunome Database (TCIA) (Charoentong et al., 2017), which was used to show the response of PRAD patients to immunotherapy. Also, Tumor Immune Dysfunction and Exclusion (TIDE) (Jiang et al., 2018) algorithm was used to assess patients’ immunotherapy response. Data for drug sensitivity analysis were obtained from Genomics of Drug Sensitivity in Cancer (GDSC) (Yang et al., 2013) by using the “oncoPredict” package. All the above data visualisations relied on R language implementation.

## 3 Results

### 3.1 Genome-wide analysis of eccDNA in prostate adenocarcinoma tumor tissues and parecancerous normal prostate tissues

**FIGURE 1:**
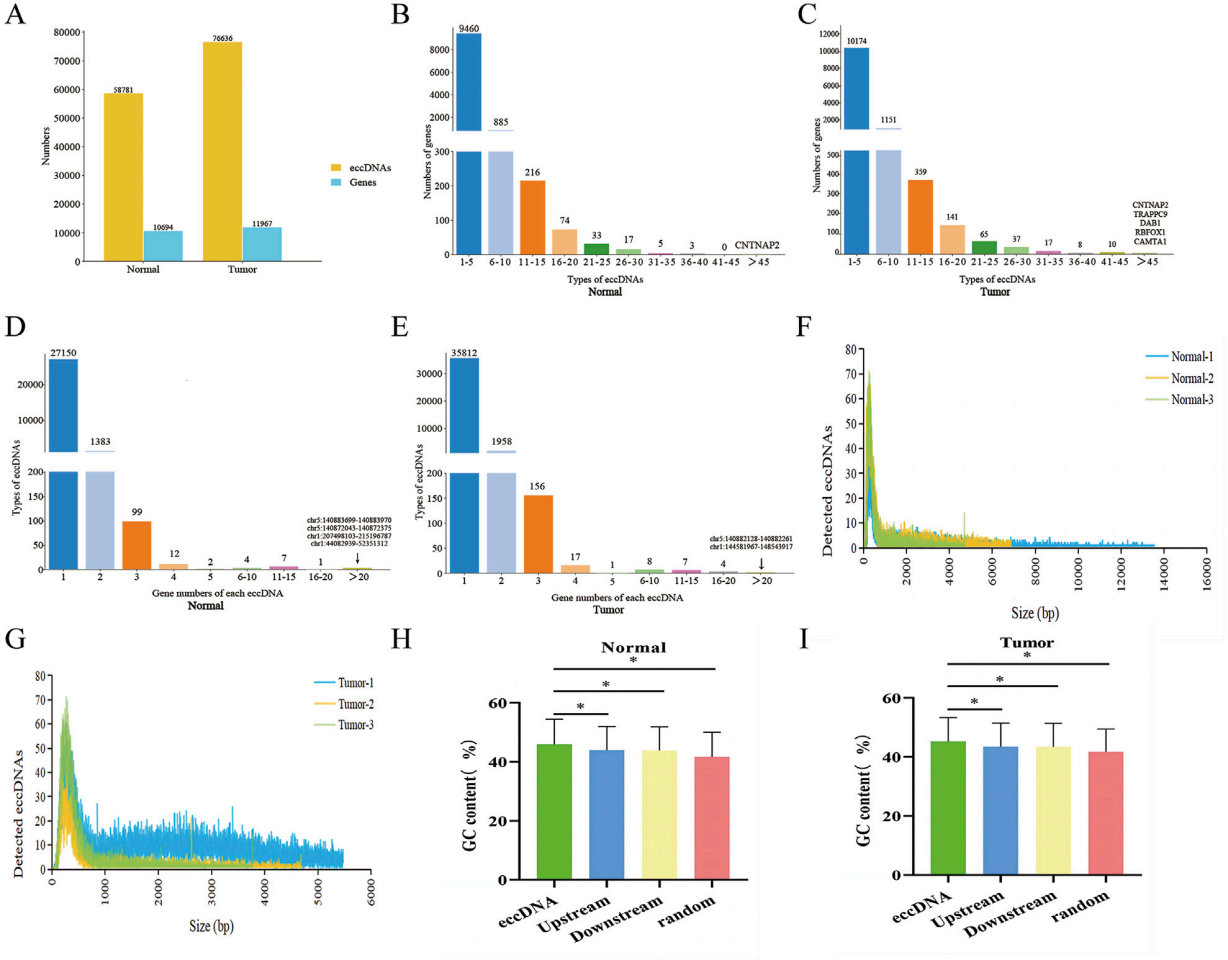
Features of eccDNAs detected in PRAD and paracancerous normal prostate tissues. **(A)** Number of eccDNA types and amplified genes from PRAD (tumor) and paracancerous normal prostate (normal) groups. **(B)** Number of genes derived from 1 to 45 and more than 45 different types of eccDNAs in normal groups. **(C)** Number of genes derived from 1 to 45 and more than 45 different types of eccDNAs in tumor groups. **(D)** Number of eccDNA types amplifying 1 to 20 and more than 20 different genes in normal groups. **(E)** Number of eccDNA types amplifying 1 to 20 and more than 20 different genes in tumor groups. **(F)** Size of eccDNA in normal groups. **(G)** Size of eccDNA in tumor groups. **(H–I)** GC contents compared to the genomic average in eccDNA, upstream, downstream and random groups from the genomic locus and regions. H, normal groups. I, tumor groups.

The eccDNA expression profiles in PRAD tumour tissues and paracancerous normal prostate tissues were obtained by eccDNA sequencing. The results showed that the tumour group contained 76,636 eccDNAsand 11,967 eGenes (Figure 1A; Supplementary Table 1), and 58,781 eccDNAs and 10,694 eGenes were identified in the normal group (Figure 1A; Supplementary Table 2). The eccDNA can encode some or all exons of a gene to affect protein expression, and different eccDNA can encode the same gene. In the normal group, we found 9,460 eGenes derived from 1-5 eccDNAs, 885 eGenes derived from 6–10 eccDNAs, 216 eGenes derived from 11–15 eccDNAs, 74 eGenes derived from 16–20 eccDNAs, 33 eGenes derived from 21–25 eccDNA derivatives, 17 eGenes were derived from 26–30 eccDNA types, 5 eGenes were derived from 31–35 eccDNA types, 3 eGenes were derived from 36–40 eccDNA types. The number of eccDNA types amplifying the CNTNAP2 gene are even more than 45, reaching up to 47 (Figure 1B; Supplementary Table 3). In the PRAD tumour group, 10,174 eGenes were detected to be derived from 1-5 eccDNA types, 1,151 eGenes were derived from 6–10 eccDNA types, 359 eGenes were derived from 11–15 eccDNA types, 141 eGenes were derived from 16–20 eccDNA types, 65 eGenes were derived from 21–25 eccDNAs, 37 eGenes were derived from 26–30 eccDNAs, 17 eGenes were derived from 31–35 eccDNAs, 8 eGenes were derived from 36–40 eccDNAs, 10 eGenes were derived from 40–45 eccDNAs, and five eGenes were derived from more than 45 eccDNA types, namely CNTNAP2, TRAPPC9, DAB1, RBFOX1 and CAMTA1 (Figure 1C; Supplementary Table 4).

At the same time, multiple coding genes could be amplified from the same eccDNA. In the normal group, there were 27,150 eccDNAs amplified 1 eGene, 1,983 eccDNAs amplified 2 eGenes, 99 eccDNAs amplified 3 eGenes, 12 eccDNAs amplified 4 eGenes, 2 eccDNAs amplified 5 eGenes, and 4 eccDNAs amplified 6–10 eGenes, 7 eccDNAs amplified 11–15 eGenes, 1 eccDNA amplified 16–20 eGenes, and 4 eccDNAs amplified more than 20 eGenes (Figure 1D; Supplementary Table 5). In the PRAD tumour group, there were 35,812 eccDNAs amplified 1 eGene, 1,958 eccDNAs amplified 2 eGenes, 156 eccDNAs amplified 3 eGenes, 17 eccDNAs amplified 4 eGenes, 1 eccDNA amplified 5 eGenes, and 8 eccDNAs amplified 6 to 10 eGenes, 7 eccDNAs amplified 11–15 eGenes, 4 eccDNAs amplified 16–20 eGenes, and 2 eccDNAs amplified more than 20 eGenes (Figure 1E; Supplementary Table 5).

In addition, we found that the size distribution of eccDNA in the normal group ranged from 10 bp to 14,000 kb (Figure 1F), and that in the tumour group ranged from 10 to 6,000 bp (Figure 1G). Both groups had an emergent peak at 300 bp (Supplementary Figures S1A–C), and there was no significant difference in the size distribution of eccDNA between the tumour and normal groups (Supplementary Figure S1D). The GC content enrichment of eccDNA in normal and tumour tissue were both significantly higher than other genomic regions (Figures 1H, I).

### 3.2 Genomic distribution of eccDNA on different chromosomes

**FIGURE 2:**
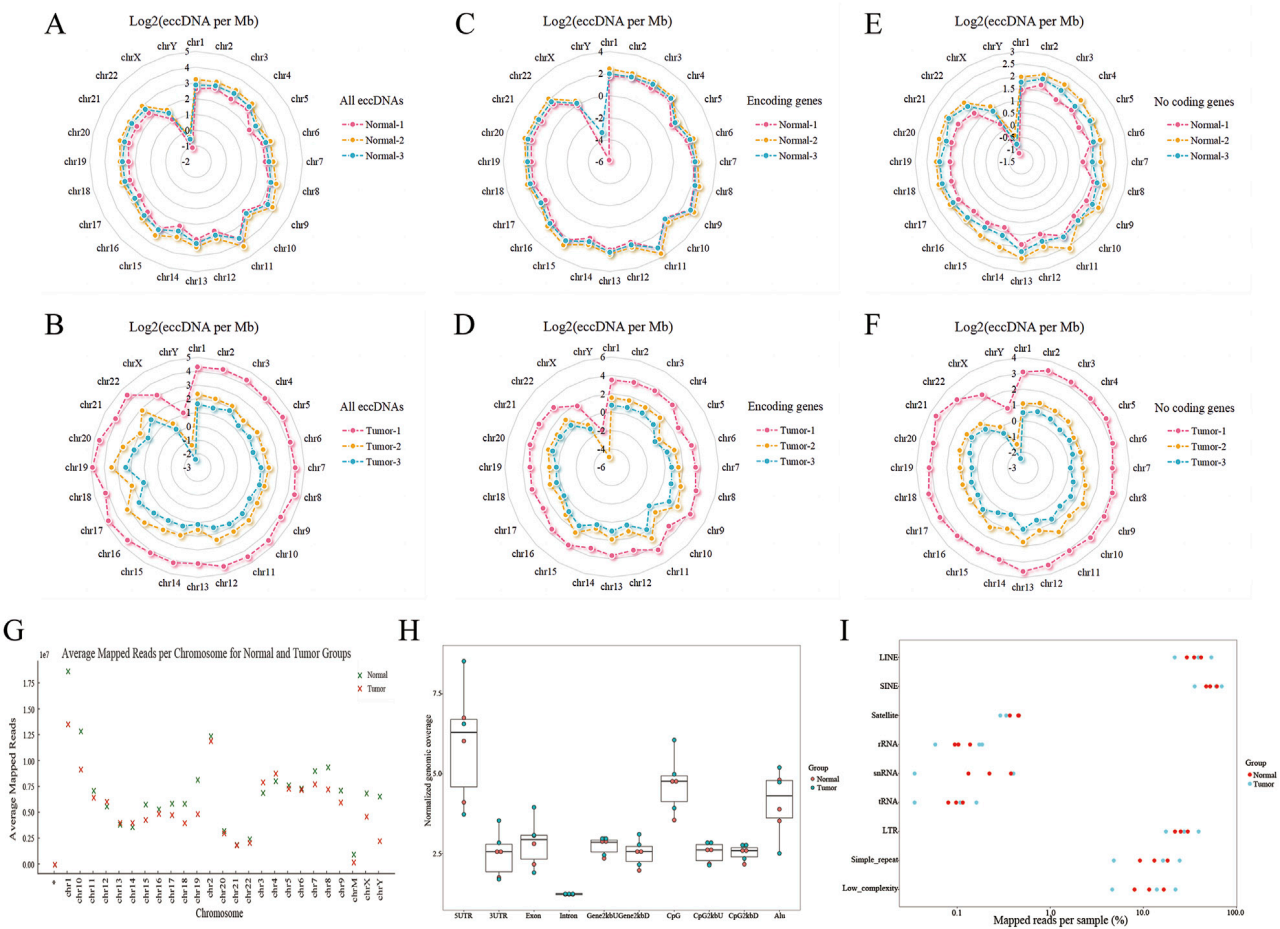
Characterization of the chromosomal and genomic distribution of eccDNAs in tumor and normal groups. **(A–F)** The radar plots showing chromosomal genome distribution of all eccDNAs, eccDNAs with and without encoding genes. A, all eccDNAs in normal group. B, all eccDNAs in tumor group. C, eccDNA with encoding genes in normal group. D, eccDNAs with encoding genes in tumor group. E, eccDNA without encoding genes in normal groups. F, eccDNAs without encoding genes in tumor groups. **(G)** EccDNA frequency counted with average mapped reads per Mb of all chromosomes in normal and tumor groups. **(H)** Genomic distributions of eccDNAs in normal and tumor groups. CpG2kbD, 2 kb downstream of CpG islands; CpG2kbU, 2 kb upstream of CpG islands; Gene2kbD, 2 kb downstream of genes; Gene2kbU, 2 kb upstream of genes. **(I)** Repetitive regions from total mapped reads for eccDNAs derived from each sample.

We further analyzed the genomic distribution of eccDNA on different chromosomes, including intact eccDNA (Figures 2A, B), eccDNA amplifying coding genes (Figures 2C, D), and eccDNA with unamplifying coding genes (Figures 2E, F). The results show that gene-rich chromosome contributed to a higher average frequency of eccDNAs per Mb than other chromosomes, such as chromosome 1, while gene-poor chromosome contributed to a lower average frequency of eccDNAs per Mb, such as chromosome Y. It suggests that regions with gene-rich are more preferentially producing eccDNA. eccDNA distribution on chromosomes between the normal group and PRAD tumour group was not significant differentiation (Figure 2G).

Finally, we explored the possible origins of eccDNAs by mapping the eccDNAs to different genomic elements (Figure 2H) and repetitive elements (Figure 2I). Notably, eccDNA was more significantly enriched in both 5′ UTR genomic region and repetitive elements, such as long interspersed elements (LINEs) and short interspersed elements (SINE), suggesting that these regions are more preferentially producing eccDNA in PRAD.

### 3.3 Differential expression of eccDNA in tumour and normal tissues

**FIGURE 3:**
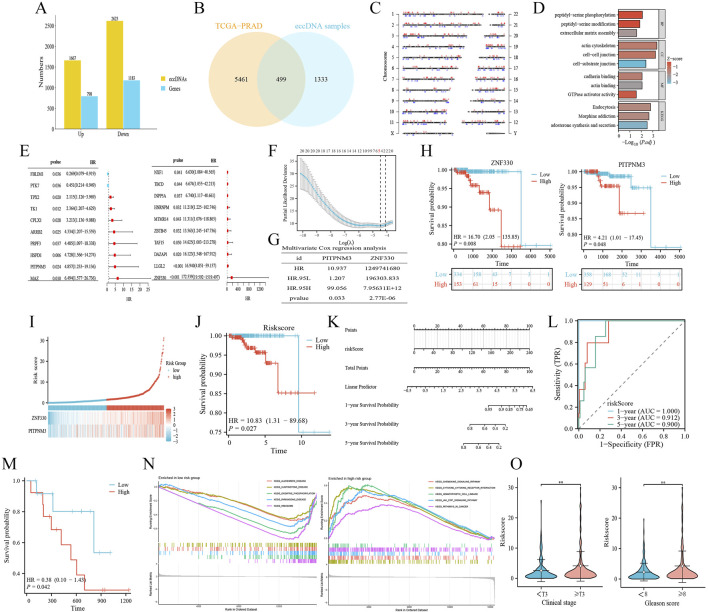
Construction of a novel PRAD risk prediction model based on differentially expressed genes amplified by eccDNA. **(A)** Number of differentially expressed eccDNAs and amplified coding genes obtained based on Circle-Seq results. **(B)** Differential eccDNA amplified coding genes and TCGA-PRAD differentially expressed genes were taken to intersect to obtain 499 eccDNA-associated differentially expressed genes (eDEGs). **(C)** Distribution of the 499 eDEGs on the chromosomes. Red, high expression; blue, low expression. **(D)** eDEGs were analyzed for functional enrichment. BP, Biological Process; CC, Cellular Component; MF, Molecular Function; KEGG, Kyoto Encyclopedia of Genes and Genomes. **(E–G)** eDEGs were sequentially subjected to different analysis. E, univariate Cox regression analysis; F, Lasso analysis; G, multivariate Cox regression analysis. **(H)** Survival analysis of ZNF330, PITPNM3. **(I)** Construction of the novel prostate risk model based on ZNF330, PITPNM3 and drawing risk factor plots. **(J)** Survival analysis of the risk model. **(K)** Prognostic nomogram based on risk score. **(L)** Time-dependent ROC curves demonstrated the predictive performance of nomogram. **(M)** Survival analysis of the validation group cBioPortal-SU2C/PCF. **(N)** GSEA functional enrichment analysis of the risk model. **(O)** The clinical correlation analysis between risk model and risk factors of PRAD.

Based on the eccDNA sequencing results, a total of 4,290 differentially expressed eccDNA were screened in PRAD tissues compared with normal tissues (Figure 3A; Supplementary Tables 6, 7). Among them, 1,667 eccDNAs were higher expressed in the tumour tissues, and these eccDNAs amplified 798 eGenes. 2,623 eccDNAs were lowly expressed and amplified 1,183 eGenes (|FC(fold change)| ≥ 2, *P* < 0.05) (Supplementary Figures S1E, F). The transcriptome data of PRAD and normal samples were obtained from the TCGA database, and 5,960 DEGs were obtained by screening (Supplementary Figure S1G). The coding genes amplified by differential eccDNA and the DEGs of TCGA-PRAD were taken to be intersected (Figure 3B), and 499 eDEGs were obtained. Further analysis of the distribution of eDEGs on chromosomes (Figure 3C) showed that eDEGs were enriched on chromosomes 1 to 22 without appear on the Y chromosome. Functional enrichment analysis of eDEGs (Figure 3D) showed that their roles were mainly focused on post-translational modification, signal transduction, and cell intercellular communication pathways.

To further explore the eKDEGs of PRAD, this study combined the TCGA-PRAD transcriptome dataset and further performed one-way Cox regression analyses (Figure 3E), LASSO analyses (Figure 3F), and multifactorial Cox regression analyses (Figure 3G) on the eDEGs. ZNF330 and PITPNM3 were finally identified as eKDEGs and independent risk factors for PRAD. ZNF330 was highly expressed and PITPNM3 was lowly expressed in PRAD (Supplementary Figure S1H), which corresponded to eccDNA sources of ZNF330^circle142141735-142142329^, PITPNM3^circle6458635-6459156^. In addition, the differential expression was validated in the GSE70770 dataset set (Supplementary Figure S1I) with consistent results.

### 3.4 Analysis of critical eccDNA ZNF330^circle142141735-142142329^ and PITPNM3^circle6458635-6459156^ at the transcriptome level

Survival analysis (Figure 3H) showed that PRAD patients with high expression of ZNF330, PITPNM3 had lower overall survival (OS), which suggested that ZNF330, PITPNM3 may be a risk factor for poor prognosis. A novel PRAD risk model was constructed by basing on these two genes, and it was found that the risk score of patients increased with higher expression of ZNF330 and PITPNM3 (Figure 3I). Patients were classified into high and low risk groups based on the risk scores, and the higher risk group had lower OS (Figure 3J), which may lead to poor prognosis. Nomograms were plotted to predict patient survival based on risk scores (Figure 3K), and the 1-year, 3-year and 5-year survival rates of patients gradually decreased with increasing risk scores. The area under the curve (AUC) of the time-dependent ROC at 1-year, 3-year and 5- years were 1.000, 0.912, and 0.900 respectively (Figure 3L), suggesting that the risk model had good predictive performance. cBioPortal-SU2C/PCF data set validated the model, and survival analysis still showed that the high-risk group was associated with poor prognosis (Figure 3M). GSEA analysis (Figure 3N) showed that the high-risk group was significantly enriched in the cytokine signalling pathway (KEGG_CYTOKINE_CYTOKINE_RECEPTOR_INTERACTION, KEGG_HEMATOPOIETIC_CELL_LINEAGE) and cancer-related pathways (KEGG_JAK_STAT_SIGNALING_PATHWAY). Meanwhile, clinical correlation analysis (Figure 3O) showed that high risk scores were positively correlated with later clinical T stage and higher Gleason scores, suggesting that PRAD in the high-risk group were more malignant. KEGG_HEMATOPOIETIC_CELL_LINEAGE) and cancer-related pathways (KEGG_JAK_STAT_SIGNALING_PATHWAY). Meanwhile, clinical correlation analysis (Figure 3O) showed that high risk scores were positively correlated with later clinical T stage and higher Gleason scores, suggesting that PRAD in the high-risk group were more malignant.

### 3.5 Risk model can reshape PRAD immune microenvironment

**FIGURE 4:**
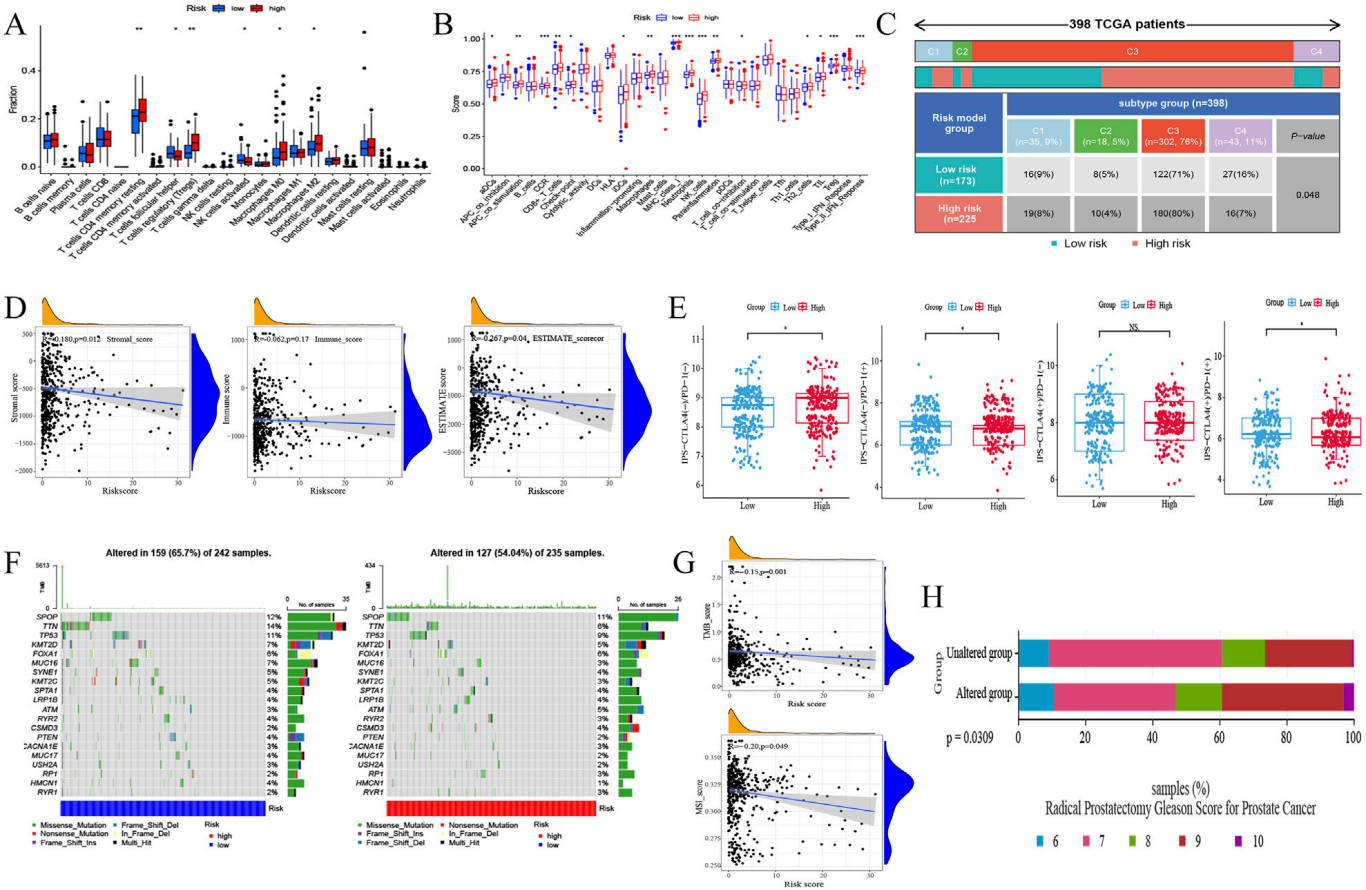
The immune landscape and mutation of risk model. **(A)** Immune infiltration analysis of risk model. **(B)** Immune function analysis of risk model. **(C)** Immunophenotyping analysis of risk model. C1, wound-healing phenotype; C2, IFN-γ dominant phenotype; C3, inflammatory phenotype; C4, lymphocyte-depleted phenotype. **(D)** Correlation of risk model with stromal score, immune score and estimate score. **(E)** Correlation of risk model and IPS score. **(F)** Waterfall plot showing the most frequently mutated genes in the risk model. **(G)** Mutation analysis of risk model. TMB, tumor mutation burden; MSI, microsatellite instability. **(H)** Correlation analysis of mutations and Gleason score ratio in PRAD under radical prostatectomy.

Quantitative analysis of the immune microenvironment was performed on the PRAD risk model based on multiple immunological algorithms. Different immune infiltration patterns were observed in patients with the high and low-risk groups. The immune infiltration analysis (Figure 4A) showed that the immune microenvironment in the high-risk group had increased levels of T cells CD4 memory resting, Macrophages M0, Macrophages M2 and Tregs, and decreased levels of T cells follicular helper and NK cells activated. Meanwhile, the infiltration abundance of some immune cells correlated with prognosis (B cells naive and Tregs infiltration were associated with poor prognosis, and Macrophages M1 and M2 infiltration were associated with better prognosis) (Supplementary Figure S2A). Single gene immune infiltration analysis of ZNF330 and PITPNM3 (Supplementary Figures S2B, C) also revealed multiple immune cell content changes. Interestingly, differential analysis of immune cell function showed that the majority of immune cells were functionally active in the high-risk group (Figure 4B), and altered immune function was associated with patient prognosis (DCs functionally active was associated with a poorer prognosis, and APC_co_inhibition, Mast_cells and Tfh functionally active were associated with a better prognosis) (Supplementary Figure S2D). In addition, there were differences of immune subtypes distribution in the risk model (Figure 4C). ESTIMATE analysis found a negative correlation between the risk model and stromal score, immune score and estimated scores (Figure 4D), suggesting that tumour purity was higher in the high-risk group. IPS scores were calculated to predict response of PRAD patients to two ICIs, anti-CTLA-4 and anti-PD-1 (Figure 4E). We found a positive correlation between the high-risk group andips-CTLA4 (−)/PD1 (−), while a negative correlation was existed between the high-risk group and ips-CTLA4 (−)/PD1 (+) and ips-CTLA4 (+)/PD1 (+) in, indicating that the PRAD model risk can influence the immunotherapy response and the high-risk group of PRAD patients had poor responses to ICIs. These results suggested that tumour immunity may play a key role in PRAD.

### 3.6 Correlation analysis of mutation with immunotherapy response in risk models

TMB and MSI are molecular markers for determining the suitability of immunotherapy for tumour patients, which also suggest genomic instability. In the risk model, we observed that the mutation rate was lower in the high-risk group (Figure 4F), and the same five most mutated genes in two risk groups were SPOP, TTN, TP53, KMT2D, and FOXA1. What’s more, the TMB and MSI scores were negatively correlated with the risk scores (TMB: R = −0.15; MSI: R = −0.2) (Figure 4G). In addition, Correlation analysis of mutations in the risk model gene set and Gleason scores (Figure 4H) showed that advanced Gleason scores were higher ratioin the mutation group in PRAD patients, which may be associated with a poor prognosis.

**FIGURE 5:**
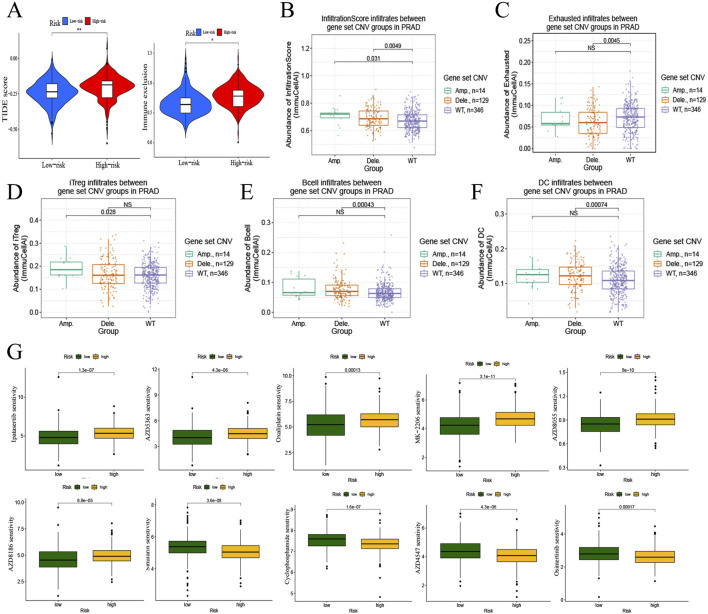
Immunotherapy and drug sensitivity to PRAD of risk model. **(A)** Correlation of risk model with TIDE score and immune exclusion. **(B–F)** Correlation analysis of mutations and immune infiltration in PRAD with gene set CNV. B, infiltrationscore infiltrates. C, exhausted infiltrates. D, iTreg infiltrates. E, B cell infiltrates. F, DC cell infiltrates. **(G)** Correlation of risk model and drug sensitivity to PRAD.

Subsequently, the TIDE score and immune exclusion was calculated for each PRAD patient based on the TIDE analysis (Figure 5A), and the risk scores were positively correlated with them. It is suggested that patients in the high-risk group are more likely to experience immune escape and poor immunotherapy. Based on the previous analysis, we found that both immune infiltration and mutation have important roles in the risk model. Therefore, we further explored the correlation between mutation and immune phenotype through the mutation profile of the gene set in the risk model. Both gene amplification (Amp) and deletion (Dele) were types of copy number variation (CNV) (Zhang et al., 2009), which were belongs to mutations. The results showed that the infiltration score was higher in the gene amplification and deletion groups compared with the wild type (WT) (Figure 5B), and the deletion group had lower exhausted abundance, whereas there was no significant difference in exhausted abundance between the wild type and amplification group (Figure 5C). The abundance of iTreg (Figure 5D) in the amplification group, and B cell (Figure 5E) and DC cell (Figure 5F) in the deletion group were higher compared to the wild type.

Drug sensitivity analysis based on the PRAD risk model gene set (Figure 5G) revealed that the high- Drug sensitivity analysis based on the PRAD risk model gene set (Figure 5G) revealed that the high-risk group decrease the sensibility of Ipatasertib, AZD5363, Oxaliplatin, MK-2206, AZD8055, and AZD8186in PRAD, and increase the sensibility of Sinularin, Cyclophosphamide, AZD4547, and Osimertinib in PRAD.

## 4 Discussion

EccDNA has long been discovered in both normal and malignant cells (Ling et al., 2021). Using circle-seq, we investigated the eccDNA profiles of PRAD and para-cancerous normal prostate tissues. It was found that ZNF330^circle142141735-142142329^ was significantly upregulated in PRAD and PITPNM3^circle6458635-6459156^ was significantly downregulated, which may be potential biomarkers in PRAD patients. We observed some conclusions that are consistent with past investigations (Kumar et al., 2017; Noer et al., 2022). For example, the size and type of eccDNA did not change significantly between PRAD tumours and normal tissues, with size distribution peaks of around 300 bp. At the same time, we discovered that the amount of amplified eGenes was identical, despite the fact that the number of eccDNA varied dramatically between these two tissues. We then investigated the matching shedding sites of eccDNA on chromosomes and discovered that there was no significant change in the chromosomal distribution of eccDNA across tissues. However, the number of eccDNA loci differed substantially among chromosomes, with chromosome 1 being the most prevalent and the Y chromosome being the least common. In the differential expression analysis of eccDNAs from PRAD tumours and normal tissues, we found that 4,290 eccDNAs were differentially expressed, and these eccDNAs amplified 1,981 eGenes. Some previous studies (Jiang et al., 2023) have shown that the expression of eccDNAs varies between cancer and normal tissues, eccDNA-amplified eGenes may not be differentially expressed. This indicates that not all differentially expressed eccDNAs play a role in disease progression. Therefore, we further explored the changes in expression levels of eccDNA-amplified eGenes in PRAD and their underlying molecular mechanisms.

This study identified eccDNA-amplified eGenes ZNF330 and PITPNM3 as key genes in PRAD. ZNF330^circle142141735-142142329^ was significantly amplified in PRAD, and ZNF330 was also consistently highly expressed in PRAD Whereas, PITPNM3^circle6458635-6459156^ was upregulated in PRAD, but PITPNM3 was lowly expressed in PRAD. This shows that eccDNA amplification may be an important, although not determining, factor influencing eGenes expression (Koche et al., 2020). Survival analysis revealed that high levels of both ZNF330 and PITPNM3 were associated with a bad prognosis, implying that they are independent risk factors for PRAD prognosis. In one study, ZNF330 was identified as a potential oncogenic factor in breast cancer (Zhang et al., 2021), and we also found that high expression of ZNF330 in PRAD led to worse clinical prognosis, which was associated with the occurrence of PRAD progressive disease (PD), DSS event (Supplementary Figures S2E, F). PITPNM3 has also been found to promote the progression of various tumours, such as breast (Zeng et al., 2023) and pancreatic cancer (Meng et al., 2015), which is an emerging therapeutic target in cancer (Torphy et al., 2022). Our work also reveals that PITPNM3 is a predictive risk factor for PRAD, and high expression of PITPNM3 is related with poor clinical stage (Supplementary Figure S2G). Thus, PITPNM3 may be related with PRAD at the transcriptional level, which requires further investigation with other samples. The above findings show that ZNF330 and PITPNM3 could be predictive indicators for PRAD.

The PRAD risk model, which was based on ZNF330 and PITPNM3, revealed that high risk was associated with a poor prognosis, and the ROC curve indicated that the model was prognostically reliable. Functional enrichment analysis revealed that the high-risk group was primarily enriched for post-translational modifications, cytokine signalling, and cancer-related pathways. More crucially, we discovered that the risk model might influence the immunological microenvironment (TME) of PRAD, directing the immunotherapy response.

Cancer development is highly correlated with the physiological state of TME (Roma-Rodrigues et al., 2019). In our study, we found that high risk was positively with the increase level of T cells CD4 memory resting, Macrophages M0, Macrophages M2, Tregs contents, and negatively with the decrease level of T cells follicular helper, NK cells in PRAD microenvironment. Tregs infiltration is also found in ZNF330 and PITPNM3 single gene immune infiltration analyses (Supplementary Figures S2B, C), which is a major mechanism of tumour immune escape, and its phenotypic and functional diversity affects its response to therapy (Kang and Zappasodi, 2023). A study of hepatocellular carcinoma (HCC) (Chen et al., 2024b) demonstrated that SOX18 overexpression mediated infiltration of Tregs and promoted HCC progression and metastasis. Macrophages M2 polarisation is a driver of tumour progression (Christofides et al., 2022), and T cells follicular helper (King, 2021) and NK cells activated (Park et al., 2023) play an important role in anti-tumour immunity. The reduction of these cells may allow tumour cells to escape immune surveillance. Meanwhile, immune cell function was active in the high-risk group. These results suggest that the risk model may be able to reshape the immune microenvironment in PRAD patients. In addition, we found that risk models influence immune subtyping, and different immune subtypes may affect the response to immunotherapy (Petralia et al., 2024).

Therefore, the impact of risk models on immunotherapy response are highly concerned. PD-1, CTLA4, TMB and MSI are all important markers for predicting the efficacy of immunotherapy. High levels of PD-1 with or without CTLA4 are generally associated with enhanced response to the corresponding targeted therapy (Yarchoan et al., 2019), and patients with high TMB and MSI scores are also more likely to benefit from immunotherapy (Valero et al., 2021). The study found that high-risk groups responded poorly to anti-PD-1 and anti-CTLA-4 therapies, which are common immune checkpoint inhibitors. This suggests that combining these immunotherapies with agents that target ZNF330 and PITPNM3, or drugs that modulate the TME, could potentially overcome resistance and improve patient outcomes. PD-1 and TMB evaluation are mainly based on the characteristics of the tumour cells, while ignoring the influence of the tumour microenvironment and immune components, such as the tumour cells themselves, the T lymphocytes and the antigen-presenting cells and other multiple immune cells’ expression (Bruni et al., 2020). Moreover, relevant factors such as tumour heterogeneity may also lead to false-negative PD-1/PD-L1 expression. Therefore, we also need to assess the tumour response to immunotherapy in terms of other factors. Higher TIDE and immunological exclusion scores in the high-risk group indicate a greater likelihood of immune escape, suggesting that these patients may require more aggressive or combination immunotherapy approaches. Additionally, the low ESTIMATE scores imply higher tumor purity, which could be factored into the development of pharmacological interventions aimed at enhancing immune infiltration and activity. These findings emphasize the need for a multifaceted approach in treating PRAD, integrating novel genetic markers, immune modulation, and personalized pharmacotherapy to improve patient prognosis and response to treatment. The research identified several drugs with varying sensitivities based on the PRAD risk model. High-risk patients showed decreased sensitivity to drugs like Ipatasertib and AZD5363 but increased sensitivity to drugs such as Sinularin and Osimertinib. This highlights the importance of personalized medicine, where drug selection is tailored based on the genetic and molecular profile of the tumor.

Although our analysis is based on precise sequencing and high-quality analyses, there are several limitations that must be acknowledged. First, putative eccDNA-amplified genes were selected and confirmed, excluding non-coding genes, which would be investigated further. Second, additional *in vitro* investigations are required to confirm the expression of ZNF330 and PITPNM3 in PRAD. Moreover, the fundamental mechanism by which eccDNA increases PRAD’s malignant tendencies is unknown and requires further investigation. Finally, although eccDNA sequencing was performed in this study, some of the analyses still originated from data in public databases, so the results await more experimental validation. We partially tested the two genes and confirmed their role in prostate cancer, while more basic research is needed in the future to better understand the role of eccDNA in PRAD.

## 5 Conclusion

In this study, we sequenced PRAD’s eccDNA and examined its size distribution, chromosomal position, and expression level. Based on eccDNA sequencing and transcriptome analysis, the important coding genes ZNF330 and PITPNM were identified as potentially transcribed from eccDNA. A unique PRAD risk model based on the two-factor combination of ZNF330 and PITPNM3 was developed, which not only predicts survival but also predicts the immunotherapy responses of PRAD patients of varying risk. These findings highlight the utility of the eccDNA-based PRAD risk model in clinical settings.

## Data Availability

The data presented in the study are deposited in the OMIX repository, accession number OMIX007219 (https://ngdc.cncb.ac.cn/omix/release/OMIX007219).

## Supplementary material

Supplementary Figure S1
**(A–D)** Comparison of eccDNA size distribution between tumor and normal groups. A, group 1. B, group 2. C, group 3. D, all groups. **(E)** Heatmap of differentially expressed eccDNAs. Red, high expression; Green, low expression. **(F)** Distribution of differentially expressed eccDNAs on all chromosomes. **(G)** Volcano plot of eccDNA amplified differentially expressed genes. Red, high expression; Green, low expression. **(H–I)** Relative RNA expression of eKDEGs (ZNF330, PITPNM3). H, TCGA-PRAD. I, validation dataset GSE70770.

Supplementary Figure S2
**(A)** Immune infiltration and survival analysis. **(B–C)** Single gene immune infiltration analysis of ZNF330 and PITPNM3. **(D)** Immune function and survival analysis. **(E–F)** Clinical correlation analysis of ZNF330. E, primary therapy outcome. F, DSS event. **(G)** Correlation analysis between PITPNM3 and clinical T stage.

Supplementary Figure S3 The expression and effect of ZNF330 in prostate cancer. **(A)** The mRNA expressions of ZNF330 and PITPNM3 in prostate normal and cancer tissues by qRT-PCR. **(B)** The protein expression of ZNF330 and PITPNM3 in prostate normal and cancer tissues from HPA database. **(C)** The mRNA expression of ZNF330 in prostate epithelial and tumor cell lines. **(D)** The mRNA and protein expression of ZNF330 with silencing ZNF330 in 22Rv1. **(E)** The effect of silencing ZNF330 and apoptosis inhibitor Z-VAD-FMK on cell proliferation of 22Rv1 with heat shock. **(F–G)** The Co-immunoprecipitation of ZNF330 with HSPA1 and HSPA8 in 22Rv1 cells.
