# Supplementary material for: Extrachromosomal circular DNAs in prostate adenocarcinoma: global characterizations and a novel prediction model
Source: Front Pharmacol. 2024 Sep 17;15:1464145. doi: 10.3389/fphar.2024.1464145 (PMC11442297; doi:10.3389/fphar.2024.1464145)
Supplement: Supplementary file 1 [file Presentation1.pdf]

Materials and methods

Quantitative Real-Time Polymerase Chain Reaction (qRT-PCR)

Prostate tissues were lysed and RNA was extracted according to the protocol of FFPE DNA/RNA kit (CWBIO, Jiangsu China). The cDNA templates were obtained through RNA reverse transcription according to the HiScript III RT SuperMix for qPCR (+gDNA wiper) kit (Vazyme, Nanjing China). The required 5'-3' primer sequences was synthesized by Sangon Biotech (Shanghai, China), what of ZNF330 are TCAGCACTCATGTCTCCGTTGT (forward) and AGCTCCATCTCCCTCTTCACCT (reverse), PITPNM3 are AATCAGTGCGGCCTA-TGGCT (forward) and AGGATCATGCGCGAGTTGTTC (reverse), and β-actin are TGGCACCCAGCACAATGAA (forward) and CTAAGTCATAGTCCGCCTAGAAGCA (reverse). The qRT-PCR reaction was performed on the StepOnePlus™ Quantitative real-time PCR system according to the ChamQ SYBR qPCR Master Mix kit (Vazyme, Nanjing China).

Protein database

The protein expression of prostate normal and tumor tissues was obtained from The Human Protein Atlas (https://www.proteinatlas.org/).

Cell lines and culture

Human prostate epithelial cell line RWPE-1 and PCa cell lines PC3, DU145, 22Rv1, LNCaP were purchased from Immocell Biotechnology Co., Ltd. (Xiamen China). They were cultured in RPMI-1640 medium (TBD, Tianjin China) with 10% FBS (Biological Industries, Israel) and 1% penicillin/streptomycin (Beyotime, Shanghai China) in the thermostatic cell incubator with 37 °C and 5% CO2.

Plasmid transfection

The plasmids were extracted with the EndoFree Plasmid Midi kit (CWBIO, Jiangsu China) to form the plasmid transfection complex for transfecting the required cells. The qRT-PCR and Western blotting experiments were performed for verification after 48 or 72 hours. The plasmids of ZNF330shnc, ZNF330sh1, ZNF330sh2, ZNF330oe, HSPA1oe, HSPA8oe were obtained from MiaoLing Bio. (Wuhan, China).

Western blotting

The collected cells were lysed and extracted as the total cellular samples. The protein concentration detection was performed by using BCA protein assay kit (Beyotime, Shanghai China) to determine the loading volume of proteins samples. The regular sequences were performed to get the PVDF membrane with protein. After primary and secondary antibody incubation, the membrane was visually exposed. The following primary antibodies were used: ZNF330 (Leading Biology, USA), β-actin (Nature biosciences, Zhejiang China), HSPA1 and HSPA8 (ABclonal, Wuhan China). The secondary antibodies HRP conjugated Affinipure Goat anti-mouse antibody and anti-rabbit antibody were purchased form Proteintech (USA).

Cell viability assay

Appropriate numbers of cells with heat shock treatment (44℃ for 2 hours) were prepared and inoculated into 96-well plates with 200μl complete medium per well. Under conventional culture, the cell viability was performed according to the protocol of cell counting kit-8 (HYCEZMBIO, Wuhan China) every 24 hours until sixth days.

Co-Immunoprecipitation (Co-IP)

The collected cells were resuspended and incubated on ice for 1 hour in NP-40 lysis buffer containing 100mM PMSF and Cocktail (Beyotime, Shanghai China). The supernatant was

rotated at 4°C with the IgG antibody solution and Protein A/G Magnetic Beads (PMB) for 2 hours to remove nonspecific protein binding. After separating the PMB with the magnetic rack, the supernatant was divided into the Input, IgG and target protein samples. The later two samples were rotated and incubated with the corresponding primary antibodies at 4°C overnight and continually rotated for 3 hours after adding PMB at next day morning. After separating the protein magnetic beads, the two supernatant of protein samples were performed with Western blotting assay along with the Input sample. These primary antibodies were used: ZNF330 (Leading Biology, USA), HSPA1, HSPA8 and IgG (Proteintech, USA).

Statistics

All data are presented as mean ± SE from three independent triplicated experiments or samples at least. GraphPad Prism 8.0.2 (GraphPad Software Inc.) was utilized for data analysis. Student's t-test was employed to compare two independent groups and One-Way ANOVA with mean values was used to compare multiple groups. The statistical significance criteria "*" represents $P < 0.05$.

Results

ZNF330 promotes the proliferation of prostate cancer cells with heat shock and is associated with heat shock proteins

We performed qRT-PCR assay on prostate normal and tumor tissues, and it was found that the mRNA expressions of ZNF330 and PITPNM3 were significantly higher in prostate cancer tissue than that in benign prostate tissue. From the analysis of HPA database, we found that the expression of ZNF330 protein in prostate cancer was significantly higher than that in benign prostate tissue, while the expression of PITPNM3 protein was not significantly different between the two tissues. Subsequently, we observed the mRNA expression of ZNF330 in prostate epithelial and tumor cell lines, and it was found that the expression of 22Rv1 was significantly increased, which make us to selected 22Rv1 for silencing ZNF330 to observe its effect on cell proliferation. By confirming the feasibility of knockdown efficiency, we selected ZNF330sh1 for silencing and found that silencing ZNF330 could significantly inhibit cell proliferation with heat shock, while the use of the apoptosis inhibitor Z-VAD-FMK could reverse the silencing effect and promote cell proliferation with heat shock. It was known that ZNF330 had immunoprecipitation with HSPA1 and HSPA8 in HEK293 cells, but whether ZNF330 has such a mechanism reaction in prostate cancer is not clear. We conducted relevant mechanism experiments and found that ZNF330 also had immunoprecipitation with HSPA1 and HSPA8 in 22Rv1 cells. Moreover, the protein expression of immunocoprecipitated in the former group was more obvious.
