# Supplementary material for: Extrachromosomal circular DNAs in prostate adenocarcinoma: global characterizations and a novel prediction model
Source: Front Pharmacol. 2024 Sep 17;15:1464145. doi: 10.3389/fphar.2024.1464145 (PMC11442297; doi:10.3389/fphar.2024.1464145)
Supplement: Supplementary file 2 [file DataSheet1.PDF]

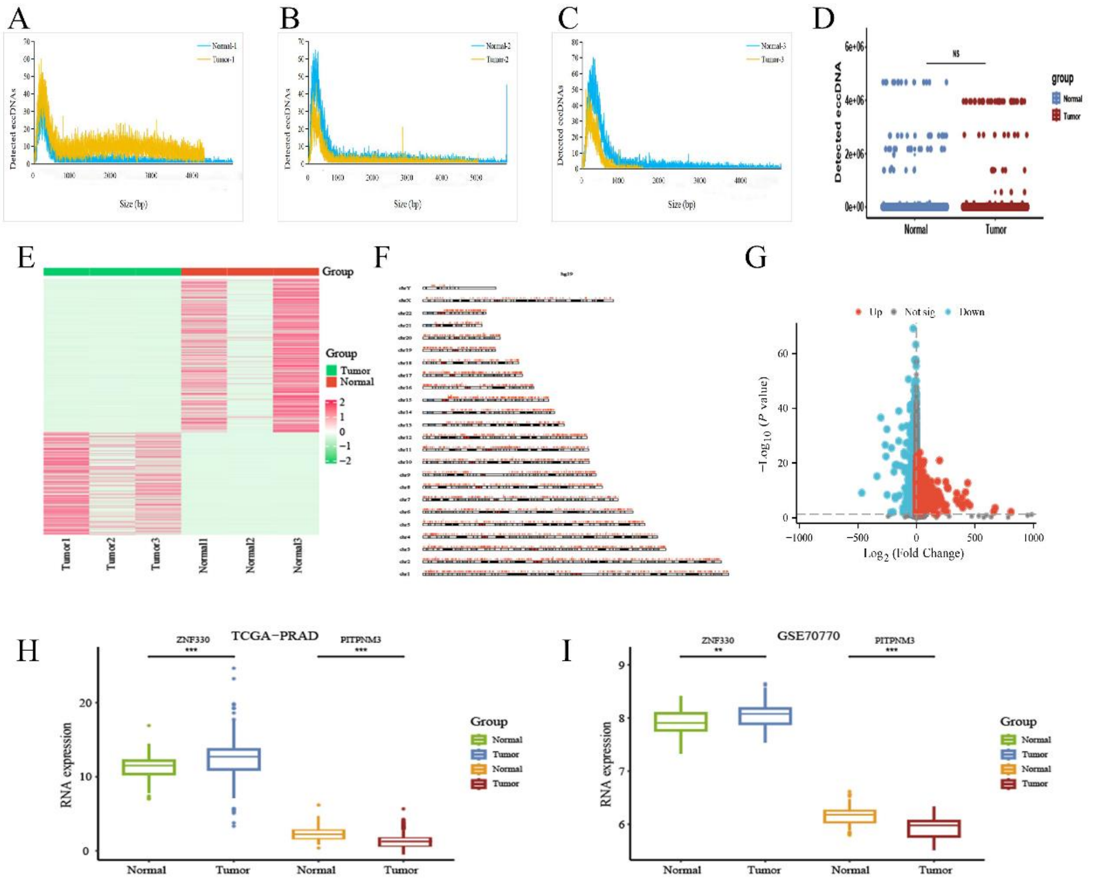

Figure S1| **(A-D)** Comparison of eccDNA size distribution between tumor and normal groups. A group 1, B group 2, C group 3, D all groups. **(E)** Heatmap of differentially expressed eccDNAs. Red, high expression; Green, low expression. **(F)** Distribution of differentially expressed eccDNAs on all chromosomes. **(G)** Volcano plot of eccDNA amplified differentially expressed genes. Red, high expression; Green, low expression. **(H-I)** Relative RNA expression of eKDEGs (ZNF330, PITPNM3). H TCGA-PRAD, I validation dataset GSE70770.

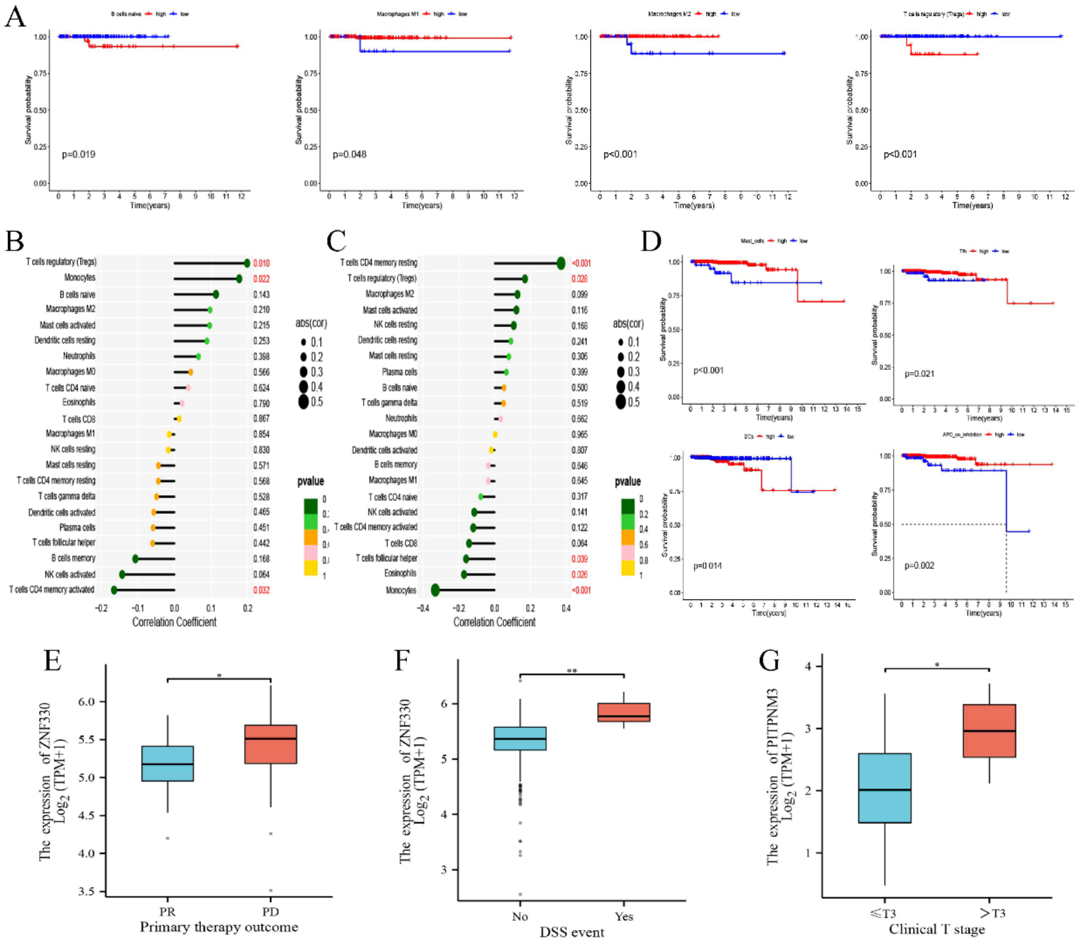

FigureS2| **(A)** Immune infiltration and survival analysis. **(B-C)** Single gene immune infiltration analysis of ZNF330 and PITPNM3. **(D)** Immune function and survival analysis. **(E-F) Clinical** correlation analysis of ZNF330. E primary therapy outcome,**F** DSS event. **(G)** Correlation analysis between PITPNM3 and clinical T-stage.

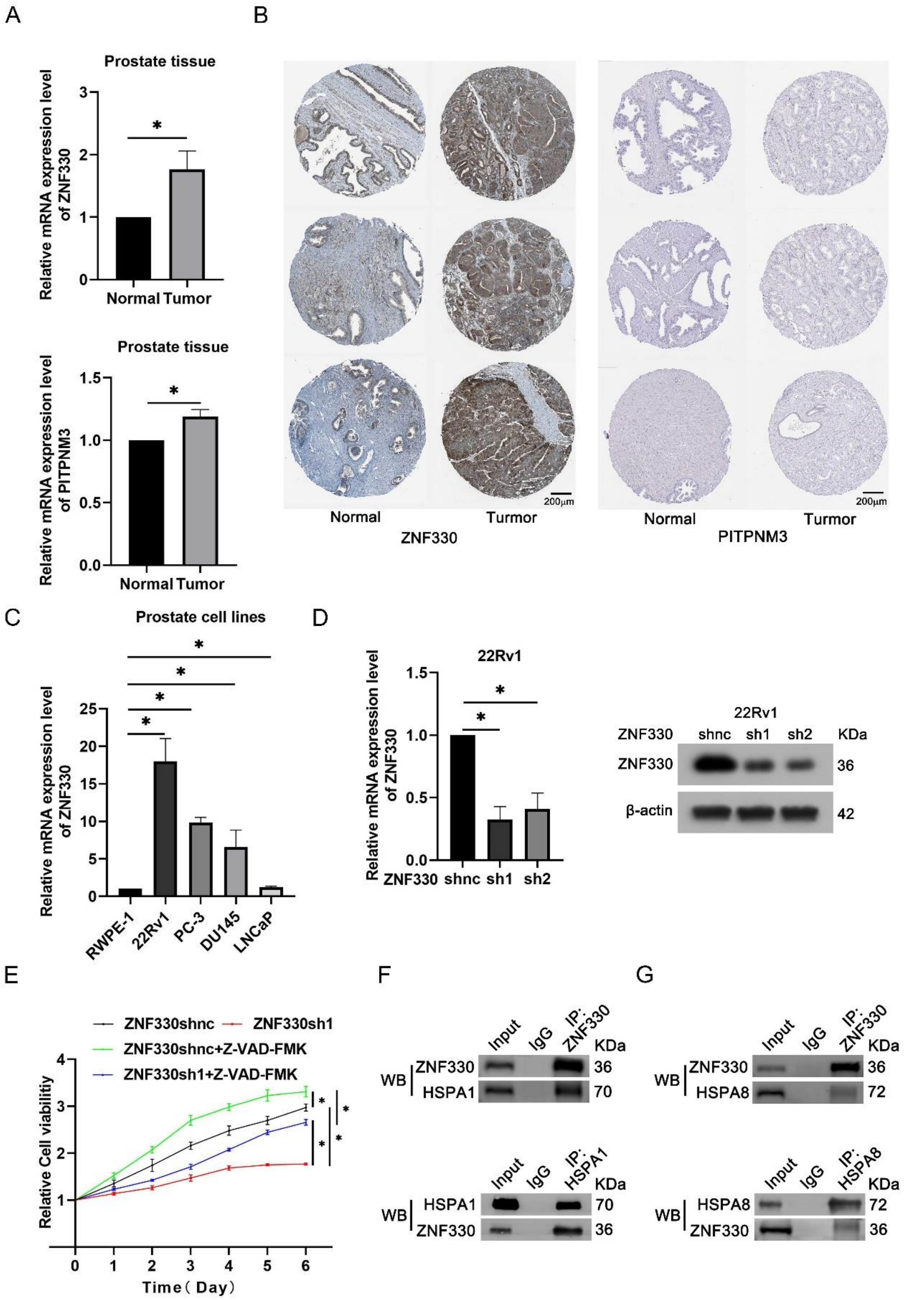

Figure S3| **The expression and effect of ZNF330 in prostate cancer.** (A) The mRNA expressions of ZNF330 and PITPNM3 in prostate normal and cancer tissues by qRT-PCR. (B) The protein expression of ZNF330 and PITPNM3 in prostate normal and cancer tissues from

HPA database. (C) The mRNA expression of ZNF330 in prostate epithelial and tumor cell lines. (D-E) The mRNA and protein expression of ZNF330 with silencing ZNF330 in 22Rv1. (E) The effect of silencing ZNF330 and apoptosis inhibitor Z-VAD-FMK on cell proliferation of 22Rv1 with heat shock. (F-G) The Co-immunoprecipitation of ZNF330 with HSPA1 and HSPA8 in 22Rv1 cells.

Table S1 | Types of eccDNA amplifying the same encoding gene in normal prostate tissue[i]

Types of eccDNAs	Numbers of genes
1	4616
2	2341
3	1304
4	736
5	463
6	295
7	218
8	167
9	110
10	95
11	56
12	62
13	43
14	29
15	26
16	25
17	17
18	10
19	11
20	11
21	8
22	8
23	5
24	7
25	5
26	5
27	3
28	6
29	2
30	1
31	3
33	1
35	1
36	1
37	2
47	1

[i] As an example, the first row indicates that there are 4616 genes amplified by one eccDNA type.

Table S2 | Types of eccDNA amplifying the same encoding gene in prostate adenocarcinoma tissue[i]

Types of eccDNAs	Numbers of genes
1	4609
2	2571
3	1456
4	921
5	617
6	359
7	296
8	218
9	140
10	138
11	103
12	79
13	71
14	61
15	45
16	48
17	29
18	25
19	24
20	15
21	17
22	16
23	12
24	11
25	9
26	10
27	11
28	8
29	6
30	2
31	5
32	1
33	4
34	2
35	5
38	3
39	4
40	1
41	3
42	1
43	1

44	3
45	2
47	1
49	1
55	2
56	1

[i] As an example, the first row indicates that there are 4609 genes amplified by one eccDNA type.

Table S3 | Gene numbers of each eccDNA[i]

Samples	Gene numbers of each eccDNA	Types of eccDNAs
Normal	1	27150
	2	1383
	3	99
	4	12
	5	2
	6	1
	7	1
	9	1
	10	1
	12	1
	14	4
	15	2
	17	1
	21	2
	45	1
	86	1
Tumor	1	35812
	2	1958
	3	156
	4	17
	5	1
	6	5
	8	1
	9	2
	11	2
	12	1
	14	2
	15	2
	18	3
	20	1
	21	1
	39	1

[i] As an example, the first row indicates that there are 27150 eccDNAs amplifying only one coding gene.
